# Overexpression of human β-defensin 2 promotes growth and invasion during esophageal carcinogenesis

**DOI:** 10.18632/oncotarget.2416

**Published:** 2014-09-05

**Authors:** Ni Shi, Feng Jin, Xiaoli Zhang, Steven K. Clinton, Zui Pan, Tong Chen

**Affiliations:** ^1^ Division of Medical Oncology, Department of Internal Medicine, The Ohio State University, Columbus, Ohio; ^2^ Division of Cardiovascular Medicine, Department of Internal Medicine, The Ohio State University, Columbus, Ohio; ^3^ Center for Biostatistics, The Arthur G. James Cancer Hospital and Richard J. Solove Research Institute, The Ohio State University, Columbus, Ohio

**Keywords:** Human β-defensin 2, esophageal squamous cell carcinoma and carcinogenesis

## Abstract

Human β-defensin 2 (HBD-2) is an antimicrobial peptide produced by mucosal surfaces in response to microbial exposure or inflammatory cytokines. Although HBD-2 is expressed in the esophagus in response to stress and infectious agents, little is known regarding its expression and functional role in esophageal carcinogenesis. In the current investigation, normal esophagus and *N*-nitrosomethylbenzylamine (NMBA)-induced precancerous and papillomatous lesions of the rat esophagus were characterized for HBD-2 encoding gene *Defb4* and protein. HBD-2 was found to be overexpressed in esophagi of rats treated with NMBA compared to animals in control group. Results of Real-time PCR, Western blot and immunohistochemistry demonstrated a positive correlation between the overexpression of HBD-2 and the progression of rat squamous cell carcinogenesis (SCC) in the esophagus. We also observed that HBD-2 is overexpressed in tumor tissues removed from patients with esophageal SCC. Moreover, *Defb4* silencing *in vitro* suppresses the tumor cell proliferation, mobility and invasion in esophageal SCC cell line KYSE-150. The results from this study provide experimental evidence that HBD-2 may play an oncogenic role in the initiation and progression of esophageal SCC and thus serves as a target for chemopreventive and therapeutic interventions.

## INTRODUCTION

Esophageal cancer shows two distinct etiological and pathological subtypes, squamous cell carcinoma (SCC) and adenocarcinoma (AC). More than 90% of esophageal cancer cases worldwide are esophageal SCC and about 5% are esophageal AC [1]. Esophageal SCC has a complex etiology. In the Western world, tobacco use and alcohol consumption are the major etiological risk factors. In nations of lower economic development, additional contributors to risk include the consumption of foods containing various mycotoxins, nutritional deficiencies, and thermal injury due to the consumption of hot beverages [2–6]. Interestingly, ethic differences are well documented in the United States with SCC predominating in African Americans over Caucasians by a ratio of 5:1 and 3:1 in males and females, respectively [7]. Unfortunately, most patients in Western nations present with advanced disease and the cure rate of esophageal SCC is less than 15% [8, 9]. Thus, opportunities existing for early diagnosis at a curable stage coupled with identification of high risk individuals for chemoprevention strategies are a high priority.

Esophageal carcinogenesis is a multistep process characterized by morphological changes from the normal epithelium to basal cell hyperplasia, dysplasia, carcinoma *in situ* and SCC. The environmental and host factors that contribute to the genetic and epigenetic changes associated with the carcinogenesis process are beginning to be elucidated [10]. Our laboratory is using the NMBA-rat model of squamous cell esophageal carcinogenesis to examine inflammatory mechanisms mediating the process and to identify targets for intervention.

Human β-defensin 2 (HBD-2), a polypeptide is expressed by epithelial surfaces and participates in host defense against bacteria, fungal and viral infections [11, 12]. HBD-2 is induced by pathogens, endotoxin (lipopolysachharide) and various proinflammatory cytokines including tumor necrosis factor-α (TNF-α), interleukin-1β (IL-1β), interferon-γ, and epidermal growth factor receptor ligands activating downstream signaling cascades involving nuclear factor kappa B (NFκB), mitogen-activated protein kinases (MAPK) and activator protein 1 [13, 14]. Some antimicrobial therapeutic agents appear to enhance the innate immune defense through inducing the expression of HBD-2 [15]. Transcriptional activity of HBD-2 encoding gene *Defb4* is correlated with antimicrobial activity [16]. The alteration of HBD-2 expression is associated with the development of various diseases related to inflammation such as psoriasis, ulcerative colitis, tuberculosis, pancreatitis, and periodontitis, and associated with clinicopathological features [17–21]. A putative role of HBD-2 in carcinogenesis is beginning to be examined but at present, it still is unclear. The down-regulation of HBD-2 was observed in patients with oral cancer and associated with the increased susceptibility to bacterial infection [19]. Similarly, patients with cervical cancer had relatively lower expression of *Defb4* [22].

Recent research progress in molecular oncology and cancer biology demonstrates that the transformation from normal epithelium to dysplasia and invasive carcinoma involves not only emerging cancer cells, but also a complex dynamic interaction between environmental components, both microbial and chemical (ex. diet or tobacco), as well as the multiple cells types in the tissue microenvironment. The objective of this study is to improve our understanding of the role that HBD-2 may play in the cascade of esophageal squamous cell carcinogenesis. In this study, we first show that HBD-2 is upregulated during carcinogen-induced esophageal SCC in the rat model. Second, we demonstrated that HBD-2 is overexpressed in human esophageal tumor tissues compared to adjacent normal tissues. Finally, we explored the functional role of HBD-2 *in vitro* and found that *Defb4* knockdown significantly inhibits esophageal carcinoma cell proliferation and mobility. Ultimately, we identified HBD-2 as novel biomarker playing a critical role in esophageal SCC.

## RESULTS

### NMBA induces esophageal carcinogenesis in rats

**Figure 1:**
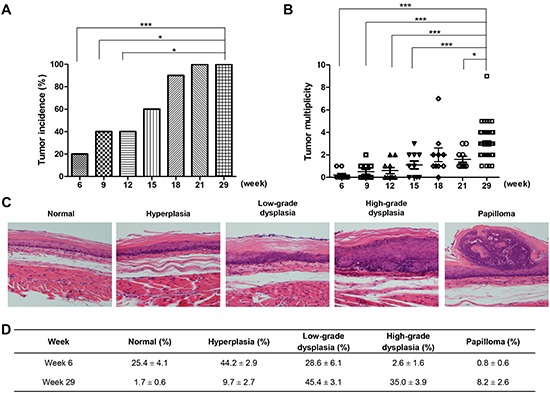
Tumor development in F344 rats treated with NMBA **(A)** tumor incidence at weeks 6, 9, 12, 15, 18, 21 and 29; **(B)** tumor multiplicity at weeks 6, 9, 12, 15, 18, 21 and 29; **(C)** H&E sections representative of normal, hyperplasia, low-grade dysplasia, high-grade dysplasia and papilloma (magnification × 200); **(D)** percentage of each histological grade of rat esophageal tissues at weeks 6 and 29. * *P* < 0.05; ** *P* < 0.01; *** *P* < 0.001.

As shown in Figure 1A, the first papillary lesions are noted one week after NMBA initiation (week 6), 2 out of 10 animals with lesion incidence increasing to 60% at week 15 and 100% by week 21. Histopathological examination of a representative sample of the tumors indicated that all were papillomas. None of the vehicle [dimethyl sulfoxide (DMSO):H_2_O] treated rats developed tumors. The tumor multiplicity also increases from week 6 to 29 (Figure 1B). Histologically, the NMBA-induced esophageal carcinogenesis is characterized by progressive morphological changes from normal squamous epithelium to basal cell hyperplasia, dysplasia (low and high grades) and papilloma (Figure 1C). At week 6, the major lesions in NMBA-treated animals were hyperplasia (44.2%), and 28.6% of lesions were low-grade dysplasia. The esophageal hyperplastic lesions progressed to dysplasia from weeks 6 to 29. At week 29, we observed 45.4% of low-grade, 35.0% of high-grade dysplasia, and normal and hyperplasia were 1.7% and 9.7% in the entire esophagus, respectively (Figure 1D).

### Microarray analysis explores HBD-2 encoding gene *Defb4* upregulated during esophageal carcinogenesis in rats

**Figure 2:**
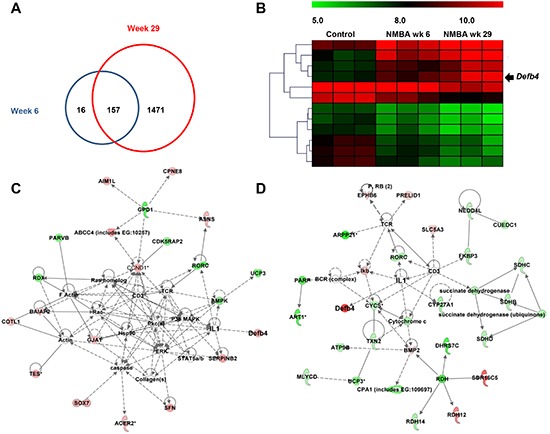
Microarray analysis **(A)** number of differentially expressed genes in rats treated with NMBA at weeks 6 (blue circle) and week 29 (red circle); **(B)** the heat map descripts the increased expression of *Defb4* in control and NMBA-treated animals from weeks 6 to 29; **(C)** the biofunction network of *Defb4* at early stage of NMBA-induced carcinogenesis (week 6); and **(D)** the biofunction network of *Defb4* at late stage of NMBA-induced carcinogenesis (week 29). Green color indicates that genes are downregulated and red color indicates that genes are upregulated during NMBA-induced esophageal carcinogenesis. Genes without green or red color are selected from literatures, which have been reported to be associated with the differentially expressed genes identified in this study. The straight lines indicate the direct interactions and dash lines indicate the indirect interactions between genes based on the data mining using IPA.

As indicated in Figure 2A, we identified 173 (125 upregulated and 48 downregulated) and 1628 (602 upregulated and 1026 downregulated) differentially expressed genes in rats treated with NMBA compared to control animals at weeks 6 and 29, respectively. Among these differentially expressed genes, 157 genes are modulated by NMBA-treatment at both weeks 6 and 29. HBD-2 encoding gene *Defb4* is one of these genes with the most significant increased fold change from weeks 6 to 29 (Figure 2B). We then used IPA software to assess the biofunction of *Defb4.* We found that it is a downstream molecule regulated by numerous genes including IL-1β, interleukin-6 (IL-6) and TNF-α at both weeks 6 and 29 (Figures 2C and 2D).

### *Defb4* mRNA and HBD-2 protein are overexpressed in NMBA-treated rat esophagus

**Figure 3:**
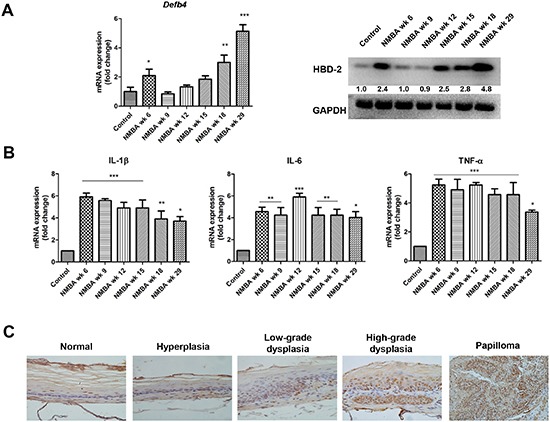
Overexpression of HBD-2 and its associated inflammatory mediators in NMBA-induced rat esophageal carcinogenesis **(A)** *Defb4* mRNA expression (left panel) and HBD-2 protein expression (right panel) in rats treated with NMBA compared to normal animals from weeks 6 to 29. The numbers under each HBD-2 blot are intensity of the blot relative to that of normal animal; **(B)** overexpression of IL-1β, IL-6 and TNF-α mRNA in rat esophagus during tumorigenesis from weeks 6 to 29; and **(C)** immunohistochemistry staining of HBD-2 in rat esophagus (magnification × 200). The values are expressed as mean; *bars*, ± SE. * *P* < 0.05; ** *P* < 0.01; *** *P* < 0.001.

We detected the expression level of *Defb4* mRNA in the epithelium of the esophagus at different time points after treatment of F344 rats with either NMBA or DMSO. The expression level of *Defb4* mRNA in NMBA-treated animals was elevated from 1.65 (week 6) - to 4.97- (week 29) fold when compared to control animals (Figure 3A, left panel). The Western blot analysis detected HBD-2 overexpression in comparable tissues (Figure 3A, right panel). We also assessed the expression of tumorigenic inflammatory mediators including IL-1β, IL-6 and TNF-α. As shown in Figure 3B, the elevated transcription of these genes in esophageal epithelium were observed from weeks 6 to 29 in NMBA-treated rats compared to normal animals. To further validate the protein expression of HBD-2, we conducted immunohistochemical analysis and found that HBD-2 protein is localized in both nuclear and cytoplasm of the epithelial cells in the basal layer of esophagus. Moreover, our data show that immunoreactivity for HBD-2 increases as the tissues progressed from normal → hyperplasia → dysplasia → papilloma (Figure 3C).

### HBD-2 protein is overexpressed in esophageal SCC in humans

**Figure 4:**
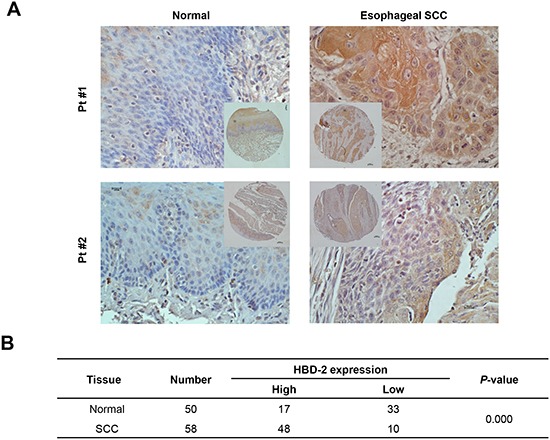
Immunohistochemistry staining of HBD-2 in human esophageal SCC **(A)** representative IHC images of HBD-2 in tumors and matched normal tissues (magnification × 200); and **(B)** IHC scores of HBD-2 staining in tumors and matched normal tissues.

To determine the expression of HBD-2 in human esophageal SCC, immunohistochemistry analysis was performed on the tissue microarrays (TMA) sample series of 58 pairs of esophageal SCC tumors and matched normal tissues (18 pairs on Cat. BC02022 and 40 pairs on Cat. ES8010). During antigen retrieval, 8 normal samples were unintentionally stripped off from the sections. Therefore, total 58 SCC and 50 normal tissues were evaluated for HBD-2 localization and distribution. As shown in Figure 4A, the immunoreactivity of HBD-2 was greater in esophageal tumors compared to their matched normal tissues (41/50 pairs). IHC scores of HBD-2 were higher in tumors than in normal tissues (*P* < 0.0001; Figure 4B).

### Overexpression of HBD-2 is observed in human SCC cell lines

**Figure 5:**
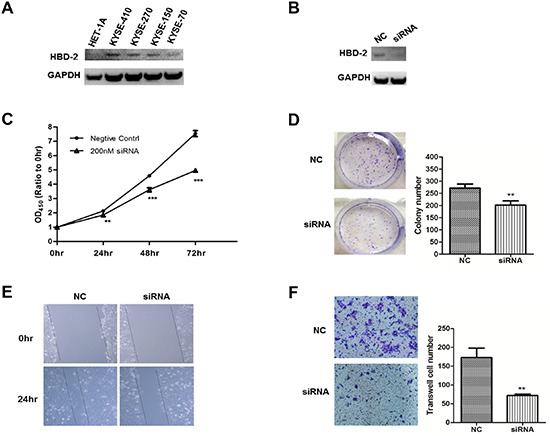
*In-vitro* functional study of HBD-2 in human esophageal SCC cells **(A)** HBD-2 protein expression in non-malignant cell HET-1A and esophageal SCC cells KYSE-410, KYSE-270, KYSE-150 and KYSE-70; **(B)** HBD-2 protein expression in KYSE-150 cells that were transfected with *Defb4* siRNA (siRNA) or scrambled siRNA (NC); **(C)** WST-1 analysis of the growth of KYSE-150 cells that were transfected with *Defb4* siRNA or scrambled siRNA; **(D)** left panel, representative photographs of colonies formed in the presence and absence of *Defb4*. Purple spots represent KYSE-150 cell colonies; and right panel, quantitative analysis of number of colonies; **(E)** scratch wound healing assay to examine the effect of *Defb4* gene silencing on KYSE-150 cell mobility; and **(F)** left panel, representative images of migratory cells that were transfected with *Defb4* siRNA or scrambled siRNA; and right panel, quantitative analysis of number of migratory cells. All the experiments were performed in triplicate. The values are expressed as mean; *bars*, ± SE. ** *P* < 0.01; *** *P* < 0.001.

To further elucidate the function of HBD-2 in esophageal SCC, we first assessed HBD-2 expression in four esophageal SCC cell lines, KYSE-70, KYSE-150, KYSE-270 and KYSE-410, and one non-tumorigenic epithelial cell line, HET-1A. We found that HBD-2 is overexpressed in all tested esophageal SCC cell lines relative to esophageal normal cell line (Figure 5A).

### *Defb4* knockdown inhibits esophageal carcinoma cell proliferation and colony formation

Western blot were conducted to assess protein expression of HBD-2 after siRNA interference KYSE-150 for 48 hours. As shown in Figure 5B, *Defb4* siRNA inhibited protein expression of HBD-2 compared to negative control cells. We conducted WST-1 assay to evaluate the effect of HBD-2 on cell proliferation of esophageal SCC. We found that the absorbance value of KYSE-150 cells transfected with *Defb4* siRNA decreased significantly at 24, 48 and 72 hours compared to negative control cells (Figure 5C). We examined the effect of siRNA *Defb4* on the capacity of KYSE-150 cells to form colonies. Knockdown of *Defb4* significantly reduced number of colonies as shown in Figure 5D.

### *Defb4* knockdown decreases esophageal carcinoma cell mobility

The scratch wound healing assay was performed to determine whether *Defb4* knockdown can suppress KYSE-150 cell mobility (Figure 5E). At 0 hour, the scratch wounds were in the similar sizes in negative control cells and cells transfected with *Defb4* siRNA. After 24 hours, comparing to control cells, the healing and migration rate of cells transfected with *Defb4 siRNA* was reduced.

### *Defb4* knockdown suppresses esophageal carcinoma cell migration

We also conducted the transwell migration assay to detect the effect of *Defb4* silencing on migration of esophageal carcinoma. We found that KYSE-150 cells transfected with *Defb4* siRNA exhibits a significant reduction in number of migratory cells compared to control cells (Figure 5F).

### HBD-2 is modulated by chemopreventive agents *in vivo* and *in vitro*

**Figure 6:**
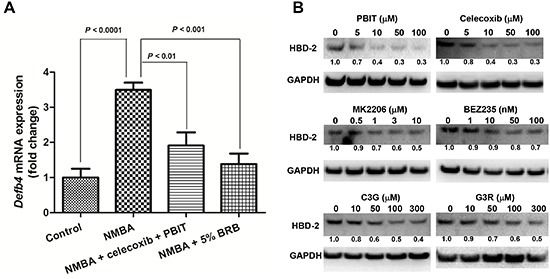
Modulation of HBD-2 by preventive agents for esophageal cancer **(A)** the expression levels of *Defb4* mRNA in rats fed with celecoxib + PBIT, 5% BRB or control diet. The values are expressed as mean; *bars*, ± SE; and **(B)** the expression levels of HBD-2 protein in KYSE-150 cells treated with different doses of PBIT, celecoxib, MK2206, BEZ235, C3G or C3R. The numbers under each HBD-2 blot are intensity of the blot relative to that of untreated control.

In our previous preclinical studies, we have identified numerous effective chemopreventive agents for esophageal cancer prevention [23–26]. To determine whether HBD-2 can be modulated by these agents, we assessed mRNA expression of *Defb4* in rat esophagus. Our data show that the combination of celecoxib, a selective cyclooxygenase-2 inhibitor, plus S,S′-1,4-phenylene-bis(1,2-ethanediyl)bis-isothiourea (PBIT), a selective inducible nitric oxide synthase inhibitor, and 5% black raspberries (BRB), significantly reduced mRNA expression of *Defb4* in rats fed experimental diet compared to those fed control diet (Figure 6A). Our data also demonstrated that the protein expression of HBD-2 in KYSE-150 is suppressed by PBIT, celecoxib, MK2206, an AKT inhibitor, BEZ235, a dual inhibitor of phosphatidylinositol-3-kinase/mammalian target of rapamycin (PI3K/mTOR), cyanidin-3-glucoside (C3G) and cyanidin-3-rutinoside (C3R) (two major anthocyanins in BRB), in a dose-dependent manner (Figure 6B).

## DISCUSSION

The NMBA-induced esophageal cancer model in rats has been proven to be a reliable and reproducible system by our group and others examining the pathogenesis of squamous cell carcinoma and potential chemopreventive interventions [23–28]. The progression of disease occurs through the same histopathologic stages of hyperplasia, dysplasia, and invasive cancer as defined in humans [23]. Thus, the application of transcriptomics to elucidating the molecular pathogenesis of the carcinogenic process in the highly controlled model may provide insight into key pathways that are targetable by chemopreventive or dietary prevention strategies. As identified by microarray analysis, one gene in particular suggested a strong correlation with cancer progression. The significant overexpression of the inflammatory protein HBD-2 and its encoding gene *Defb4* was observed following exposure to NMBA in rats.

HBD-2 is known to be induced by bacterial pathogens or pro-inflammatory mediators in many epithelial cell types [29]. It has direct anti-microbial potential and has been shown to possess chemotatic activity in immune cells including dendritic cells, T- cells, monocytes and also orchestrates cytokine production by these infiltrating cells [30]. Inflammatory mediators, IL-1β and transforming growth factor-β1, both associated with pro-carcinogenic chronic inflammation, and virus, such as human papilloma virus, can significantly induce the expression of HBD-2 [31, 32]. IL-1β is identified as an anchor of inflammation-cancer network in esophagus SCC in rats with zinc deficiency (ZD) [33, 34]. It is reported to be significantly overexpressed in esophageal cancer [35]. In this study, we observed an overexpression of IL-1β in NMBA-treated animals compared to control animals. The microarray analysis in NMBA and ZD rat model of esophageal SCC is compared and summarized in Tables S1 and S2. Some molecules involved in IL-1β/HBD-2 regulation, such as NFκB and MAPK, have been activated in esophageal SCC [36–38]. Moreover, numerous studies reported that HBD-2 serves as an immune modulatory factor in prostate cancer and its overexpression has been observed in tissues with inflammation [39]. HBD-2 plasmid transfection can induce proliferation, increase mobility and decrease apoptosis of esophageal SCC KYSE-150 cells (Figure S1). In this study, we found that NFκB is activated (Figure S2A) and the transcription of some proinflammatory cytokines including IL-6 and TNF-α, which are regulators of HBD-2, are enhanced by NMBA in rats. We think that HBD-2 plays a critical role in esophageal SCC, at least in part, due to its involvement in alterations of inflammatory microenvironment. To demonstrate the modulation of HBD-2 in esophageal SCC in humans, we conducted immunohistochemistry analysis of HBD-2 in tumor tissues in esophageal SCC patients. Our data show that the production of HBD-2 in esophageal epithelium is higher in tumor specimens relative to non-malignant tissues in humans.

Since HBD-2 is mainly expressed in a variety of epithelia and plays an important role in antimicrobial innate immune response against a broad spectrum of pathogens including bacteria, fungi and viruses, numerous studies have been conducted to explore the functional role of HBD-2 in various diseases. The results till date, however, have been contradictory and limited, especially in epithelial malignancies. This is the first study to elucidate the cellular mechanisms of HBD-2 in esophageal cancer. Our *in-vitro* investigations show that knockdown of HBD-2 encoding gene *Defb4* suppresses esophageal carcinoma cell proliferation and metastasis. In addition, activations of NFκB and its upstream regulators, AKT and extracellular-signal-regulated kinase (ERK), are suppressed by HBD-2 knockdown (Figure S2B). NFκB subunits have been reported to be essential for the transcription activation of HBD-2 promoter [40]. We think that NFκB is clearly associated with the overexpression of HBD-2. Moreover, there may be a loop between NFκB and HBD-2. The overexpression of HBD-2 may contribute to esophageal SCC carcinogenesis by the feedback regulation of NFκB. As reported by Blagosklonny, under growth-limiting condition, such as inflammation and hypoxia, cells can become adaptation to these conditions by activating cell proliferation and suppressing apoptosis, which is called oncogenic type resistance [41]. Our study showed that HBD-2, which is involved in inflammation, simultaneously activates NFκB and provides oncogenic properties to the esophageal epithelial cells, and thus promotes tumor growth. Further studies are warranted for the understanding of cross-talk network among NFκB, HBD-2 and key proinflammatory mediators in cell proliferation, tumor migration and invasion.

In the current study, we further determine whether HBD-2 could serve as an intermediate biomarker to assess the efficacies of preventive agents for esophageal cancer prevention. We thus assessed its alteration in rat esophagi and human esophageal cancer cells which were treated with preventive agents. BRB, celecoxib and PBIT were reported to suppress tumor development in rat esophagus in our previous studies [23–26]. MK2206 and BEZ235 have been shown to inhibit tumor formation in xenograft mouse model (Chen unpublished data). C3R and C3G are major anthocyanins in BRB. We found that NMBA-treated rats and human esophageal SCC cells, which have relatively lower expression of HBD-2, have better response to treatment agents. HBD-2, therefore, could serve as an intermediate biomarker, not only to predict the precancerous growth in esophagus, but also to monitor the efficacies of preventive or therapeutic interventions. Our studies *in vivo* and clinical studies warrant further investigations of the role of HBD-2 in esophageal cancer.

In conclusion, using the NMBA multistage esophageal carcinogenesis rat model, we found that HBD-2 was significantly upregulated during progression of the malignant process. In addition, we detected overexpression of HBD-2 in human esophageal cancer specimens. Moreover, silencing *Defb4* suppresses cell proliferation and metastasis in *vitro*. Our study provides a systemic examination of HBD-2 in cell systems, *in vivo* models and human tissues of esophageal SCC, each having specific advantages for biomarker research. Information from this study may be helpful in developing chemopreventive/therapeutic agents targeting HBD-2.

## METHODS

### *In vivo* model of esophageal SCC

Animal care and experiments were approved by the Institutional Animal Care and Utilization Committee in The Ohio State University. One hundred and forty Fisher 344 rats, 4-5 weeks old, were obtained from Harlan Sprague Dawley **(**Indianapolis, IN) and housed 3 per cage under standard conditions (20 ± 2°C, 50 ± 10% relative humidity, and 12 hours light/dark cycles). After two weeks of acclimation to the animal facility, the rats were administered s.c. injections of 0.2 ml of either NMBA (0.3 mg/kg body weight) or a 1:4 mixture of (DMSO):H_2_O (the solvent for NMBA) 3 times per week for 5 weeks. Ten esophagi were collected from each group (NMBA-treated and DMSO-treated) at 6, 9, 12, 15, 18, 21 and 29 weeks following initiation of NMBA treatment. As described previously [24], the esophagus was cut longitudinally into two parts. Half of each esophagus was fixed in 10% neutral buffered formalin for 4 hours, and then transferred to phosphate buffered saline. These tissues were then cut into three segments and embedded in paraffin. The other half of esophagus was stripped of submucosal and muscularis layers and frozen in liquid nitrogen.

### Microarray analysis and ingenuity pathway analysis (IPA)

The total RNA was extracted from frozen rat esophageal tissues using RNeasy Mini Kit (Qiagen, Valencia, CA) according to the manufacturer's protocol. The integrity of RNA was determined by Agilent 2100 bioanalyzer (Agilent Technologies, Santa Clara CA). The RNA samples with RNA Integrity Number (RIN) above 8 were used for microarray analysis. The RNA pools (3 individual samples with equal quality), which were collected from control animals, NMBA-treated animals at weeks 6 and 29, were hybridized with Affymetrix Rat Genome 230 2.0 Array (Affymetrix). Microarray data is accessible at NCBI GEO database (GSE60493). For gene-by-gene statistical analysis, parametric tests were performed to compare differences in gene expression among groups. The False Discovery Rate (FDR) was employed using Benjamini-Hochberg procedure for multiple testing to result the significance. IPA software (http://www.ingenuity.com) was used to analyze probable network and biofunction.

### Esophageal tissue microarray

Esophageal tissue microarrays (TMA) were purchased from US BiomaxInc (Rockville, MD). Microarray panel 1 (Cat. BC02022) contains 18 cases and 54 cores (1 malignant tumor, 1 adjacent tissue and 1 normal tissue for each case – 3 × 18). Microarray panel 2 (Cat. ES8010) contains 40 cases and 80 cores (1 malignant tumor and 1 normal tissue for each case – 2 × 40).

### Real-time quantitative PCR

The total RNA was extracted from frozen rat esophagus tissues using RNeasy Mini Kit (Qiagen, Valencia, CA) according to the manufacturer's protocol.cDNA was transcripted with High capacity cDNA Reverse transcription Kit (Applied Biosystems, Foster City, CA). The expression of *Defb4* was determined using Taqman assays: *Defb4* (Rn02532184_g1), using GAPDH as reference gene (Rn01775763_g1). The expression of IL-1β, IL-6 and TNF-α was determined using SYBR Green. Primers for the genes of interest were: IL-1β (Forward 5′-TGTATGCTACCATCTGGCTTCGG-3′, Reverse 5′-GTTTGGAACAGTCGCTCGTCATC-3′), IL-6 (Forward 5′-TTGGGTCTTGTTAGCCTAGTC-3′, Reverse 5′-TGTGCAGTCCCAGTGAGGAAC-3′), TNF-α(Forward 5′-TGTATGCTACCATCTGGCTTCGG-3′, Reverse 5′-GTTTGGAACAGTCGCTCGTCATC-3′) and GAPDH (Forward 5′-TATTGGGCGCCTGGTCACCA-3′, Reverse 5′-CCACCTTCTTGATGTCATCA-3′). Taqman arrays, TaqMan® Fast Advanced Master Mix and Fast SYBR Green Master Mix were purchased from Applied Biosystems (Foster City, CA). Each sample was analyzed in triplicate on ABI PRISM 7900 (Applied Biosystems).

### Western blot analysis

Proteins were extracted from tissues or cells using Cell Lysis Buffer (Cell Signaling Tech., Beverly, MA). Protein concentration was determined using the DC Protein Assay (Bio-Rad, Hercules, CA). The Western blot was carried on XCellSureLock® Mini-Cell and XCell II™ Blot Module (Invitrogen, Carlsbad, CA). The primary antibody for HBD-2 and GAPDH were purchased from Abcam (Cambridge, MA) and Cell Signaling Tech, respectively. The protein bands were visualized in ChemiDocXRS^+^ Systems (Bio-Rad).

### Immunohistochemistry

The slides containing rat or human esophageal tissues were deparaffinized with histoclear and rehydrated in graded ethanol (100–70%). Sections were incubated with 3% hydrogen peroxide, casein and goat serum, adivin and biotin, and then incubated with HBD-2 antibody (rat tissues: Bioss, Woburn, MA; human tissues: Abcam, Cambridge, MA) followed by goat anti-mouse biotinylated immunoglobin link, and strepavidin–horseradish peroxidase label. Finally, the sections were developed with diaminobenzidine. Reagents were supplied by BioGenex (Fremont, CA). The immunohistochemistry staining on TMAs was analyzed semi-quantitatively. In brief, the final score was obtained by multiplying staining intensity (0 = negative, 1 = weak, 2 = moderate and 3  = strong) and percentage of positive-staining cells (0 = < 10%, 1 = 10–25%, 2 = 25–50% and 3 = > 50%) [42].

### Cells and cell treatment

KYSE-70, KYSE-150, KYSE-270 and KYSE-410 cell lines were purchased from DSMZ (Braunschweig, Germany). KYSE-70 and KYSE-150 were cultured in 1:1 mixture of RPMI-1640 medium and Ham's F12 Medium + 2% fetal bovine serum (FBS). KYSE-270 and KYSE-410 were cultured in RPMI-1640 medium with 10% FBS, in a 37°C, 5% CO_2_ environment. HET-1A cells were obtained from American Type Culture Collection and cultured in LCH medium. To assess whether previously identified chemopreventive agents for esophageal SCC could modulate HBD-2, esophageal carcinoma cells were treated with different doses of PBIT, celecoxib, MK2206, BEZ235, C3G or C3R. Protein was extracted from the cells after 24 hours and used for Western blot analysis.

### Oligonucleotides and cell transfection

Fifty nM siRNA sequence against *Defb4* and scramble sequence were transfected with Lipofectamin LTX (Life Technologies, Grand Island, NY) to KYSE-150 cells at 50% confluent in a 6-wells plate. Proteins were extracted from cells after transfected with *Defb4* siRNA for 48 hours to confirm the gene silencing. The capabilities of proliferation and metastasis of cells transfected with *Defb4* siRNA were detected by the following assays.

### Cell proliferation assay

The proliferation of cells was assessed using the WST-1 kit (Cayman, Ann Arbor, Michigan). After transfected with *Defb4* siRNA, KYSE-150 cells were seeded in a 96-wells plate at a density of 1500 cells/well in 100 μl medium for 4 days. Cells were incubated with 10μl WST-1 reagent at 37°C in a 5% CO_2_ incubator for 2 hours. Cell viability was then detected at 0, 48 and 72 hours by plate reading at 550 nm using Omega Microplate Reader (BMC Labtech., Offenburg, Germany).

### Colony formation assay

After transfected with siRNA, 2000 KYSE 150 cells were seeded in each 6-well plate and incubated for 10 days. Colonies were fixed and stained with crystal violet. The total number of colonies consisting of more than 50 cells was counted in each well.

### Wound-healing assay

After transfected with siRNA, KYSE-150 cells were wounded by a 10 μl sterile pipette tip, and washed in PBS to remove cellular debris and allowed to culture for 24 hours. Cells were photographed at 0 and 24 hours after wounding. The wound closure was calculated after 24 hours.

### Migration assay

After transfected with siRNA, KYSE 150 cells (5×10^4^) suspended in serum-free 1:1 mixture of F12 and RPIM 1640 were added to the upper chamber (6.5-mm diameter, 8-μm pore size, Corning), and the chamber was placed in 24-well dishes containing with the same medium with 5% FBS. Migration assays were carried out for 24 hours, and then cells were fixed with 100% methanol. Cells were stained with crystal violet staining solution. Cells on the upper side of the insert were removed with a cotton swab. Three randomly selected fields (10 × objective) were photographed, and the migrated cells were counted. The migration was expressed as the average number of migrated cells in a field.

### Statistical analysis

The histological grade of esophageal tissues, mRNA expression levels of *Defb4*, IL-1β, IL-6 and TNF-α, protein expression level of HBD-2, and data from assays for cell proliferation, wound healing, colony formation and migration were compared by Student's *t*-test. All statistical analysis was carried out using GraphPad Prism 5.0. Differences were considered statistically significant at *P* < 0.05. All *P* values were two-sided.

## SUPPLEMENTARY FIGURES AND TABLES


