# Supplementary material for: Overexpression of human β-defensin 2 promotes growth and invasion during esophageal carcinogenesis
Source: Oncotarget. 2014 Sep 5;5(22):11333–44. doi: 10.18632/oncotarget.2416 (PMC4294379; doi:10.18632/oncotarget.2416)
Supplement: Supplementary file 1 [file oncotarget-05-11333-s001.pdf]

SUPPLEMENTARY FIGURES AND TABLES

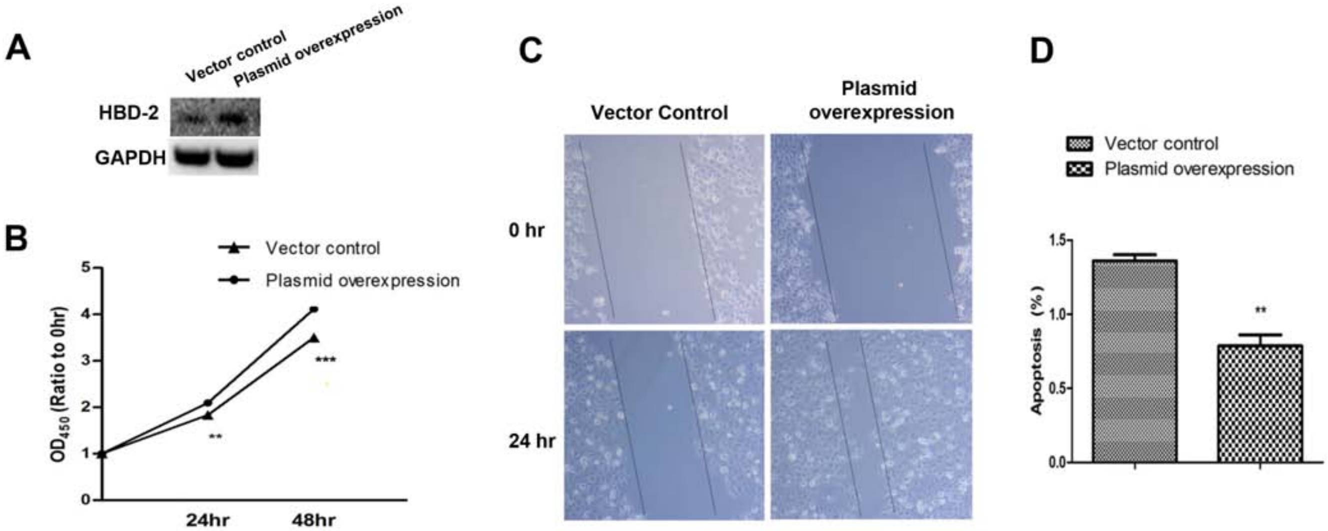

Supplementary Figure S1: *In-vitro* functional study of HBD-2 overexpression in human esophageal SCC cells. (**A**) HBD-2 protein expression in KYSE-150 cells transfected with HBD-2 plasmid; **(B)** WST-1 analysis to detect proliferation of KYSE-150 cells with HBD-2 overexpression; **(C)** scratch wound healing assay to examine cell mobility; and **(D)** flow cytometry to assess apoptosis of KYSE-150 cells transfected with HBD-2 plasmid. The values are expressed as mean; bars, ± SE; ** $P < 0.01$; *** $P < 0.001$

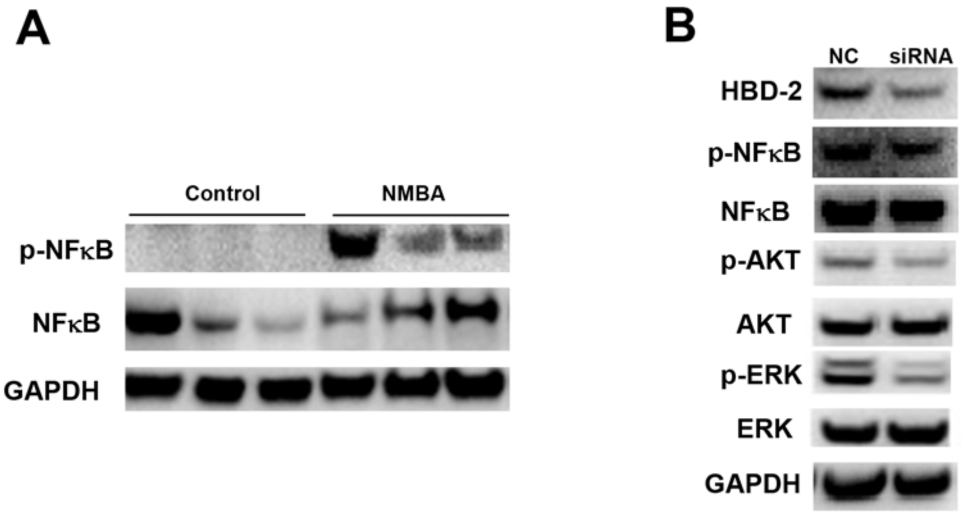

Supplementary Figure S2: Western blot analysis of HBD-2, NFκB, AKT and ERK in rat esophagus and human esophageal SCC KYSE-150 cells. (A) NFκB is activated by NMBA treatment in the rat esophagus; and **(B)** *Defb4* siRNA suppresses activations of NFκB, AKT and ERK in KYSE-150 cells.

Supplementary Table S1. Differentially expressed genes identified in both NMBA- and zinc-deficiency-induced rat esophageal SCC at the early stage of tumorigenesis.

Gene symbol	Affymetrix probe ID	ZD animal model		NMBA animal model	
		Fold change	q-value (%)	Fold change	*P*-value
**Down-regulated genes**					
Ak1	1389185_at	0.41	0	0.36	0.000982768
Irx3_predicted	1390776_at	0.16	0	0.36	0.000638621
Ppp1r3c	1373108_at	0.23	0	0.25	0.000146778
Gpd1	1371363_at	0.44	0.01	0.35	0.000164274
**Up-regulated genes**					
Slc2a1	1370848_at	2.04	0.11	2.03	0.000759009
Baz1a_predicted	1377727_at	2.09	0	2.01	0.000317027
Cotl1_predicted	1388596_at	2.25	0	2.05	0.000938934
Cpne8_predicted	1391830_at	2.27	0.07	2.07	5.69E-05
Lgals3bp	1387946_at	2.35	0.07	2.17	2.18E-05
Id1	1387028_a_at	2.39	0	2.33	1.36E-05
LOC683295	1371757_s_at	2.39	0.07	9.42	0.000672653
Ocm	1370477_at	2.56	0.04	6.08	9.17E-06
RT1-Bb	1371033_at	2.59	0.08	2.96	0.000411571
Ptprz1	1368350_at	2.68	0.03	2.36	0.000188028
Akr1b8	1370902_at	2.72	0.01	2.03	0.000891636
Scarb1	1367855_at	2.72	0	2.02	0.000140649
Asns	1387925_at	3.27	0.03	2.13	5.96E-05
Anxa1	1394451_at	5.05	0	2.56	0.000520291
Slfn3	1387134_at	5.82	0	3.85	0.00034384

Supplementary Table S2. Differentially expressed genes identified in both NMBA- and zinc-deficiency-induced rat esophageal SCC at the late stage of tumorigenesis.

Gene symbol	Affymetrix probe ID	ZD animal model		NMBA animal model	
		Fold change	q-value (%)	Fold change	*P*-value
**Down-regulated genes**					
Acadm	1367702_at	0.46	0.06	0.43	3.15E-06
Gsta3	1367774_at	0.47	0.29	0.49	2.29E-05
NA	1373167_at	0.11	0.00	0.21	9.91E-06
Pvalb	1370214_at	0.14	0.00	0.31	0.000769
Pdlim3	1370291_at	0.24	0.00	0.30	0.000778
Dmn	1372658_at	0.26	0.00	0.36	4.87E-05
Ryr3	1398479_at	0.28	0.00	0.24	3.71E-06
Flnc_predicted	1388496_at	0.29	0.00	0.30	0.00046
Ubl3	1383223_at	0.30	0.00	0.50	0.000675
Hspb8	1388721_at	0.33	0.05	0.32	0.000187
Clu	1367784_a_at	0.34	0.05	0.43	0.00026
Lmod1_predicted	1374237_at	0.34	0.05	0.28	9.56E-07
Dtna_predicted	1380964_at	0.34	0.00	0.49	2.57E-06
Hspb8	1387282_at	0.34	0.05	0.21	0.00035
NA	1388935_at	0.35	0.05	0.46	2.31E-05
RGD1565759_predicted	1383704_at	0.36	0.05	0.21	1.44E-06
Egln3	1368174_at	0.37	0.05	0.47	8.15E-08
Nexn	1370854_at	0.38	0.05	0.26	3.2E-05
Dmpk_predicted	1373915_at	0.38	0.05	0.33	1.68E-05
Dscr1l1	1389066_at	0.39	0.07	0.26	2.98E-07
Irx3_predicted	1390776_at	0.41	0.14	0.18	8.5E-06
Plekhc1	1398327_at	0.41	0.06	0.45	2.49E-05
Ndrg2	1387121_a_at	0.42	0.06	0.28	0.000108
LOC684050	1373368_at	0.43	0.56	0.47	0.000195
NA	1371679_at	0.44	0.07	0.33	0.000105
S100b	1386903_at	0.44	0.56	0.31	1.18E-06
Tpm1	1370288_a_at	0.45	0.14	0.45	0.00037
Rapgef4	1371081_at	0.45	0.14	0.50	1.12E-05
RGD1305176_predicted	1398245_at	0.45	0.16	0.27	0.000462
Asb2	1372280_at	0.46	0.09	0.25	7.88E-06

Gene symbol	Affymetrix probe ID	ZD animal model		NMBA animal model	
		Fold change	q-value (%)	Fold change	*P*-value
RGD1562983_predicted	1372684_at	0.46	0.11	0.42	1.27E-05
Plekhc1	1384182_at	0.47	0.29	0.42	1.21E-06
RGD1564128_predicted	1385682_at	0.47	0.56	0.38	5.31E-05
Atp1b1	1367814_at	0.48	0.29	0.23	8.75E-05
Nrep	1371412_a_at	0.48	0.29	0.29	2.87E-06
RGD1560011_predicted	1372403_at	0.48	0.16	0.39	3.44E-05
NA	1388866_at	0.48	0.29	0.22	1.13E-05
Ivd	1370232_at	0.49	0.09	0.48	2.34E-06
Atp1b1	1386937_at	0.49	0.29	0.28	0.000177
Igfbp5	1370960_at	0.50	0.70	0.24	3.37E-05
RGD1307524_predicted	1389007_at	0.50	0.07	0.40	3.6E-07
**Up-regulated genes**					
S100a9	1387125_at	2.00	0.56	8.09	7.82E-06
Junb	1387788_at	2.20	0.56	2.28	5.64E-06
Myo5a	1368450_at	2.21	0.07	2.49	1.54E-07
Emb	1368541_at	2.26	0.16	2.62	1.94E-07
LOC683586	1390771_at	2.32	1.53	2.38	0.000989
Es2	1371100_at	2.56	0.06	2.00	0.000362
Mcm3_predicted	1372406_at	2.64	0.03	2.04	0.000163
Timeless	1368522_at	2.66	0.03	2.21	0.000855
Mcm6	1371074_a_at	3.26	0.00	2.43	0.000709
Ifitm1_predicted	1372013_at	3.37	0.00	2.16	0.000248
Sult1b1	1387314_at	3.38	0.03	2.98	0.000742
Mad2l1_predicted	1398602_at	3.72	0.00	2.04	0.000129
RGD1309350_predicted	1377660_at	3.77	0.00	2.31	4.32E-06
Anxa1	1394451_at	5.05	0.00	2.62	0.000374
Ca2	1386922_at	32.09	0.00	2.35	1.39E-05
